# Estimating the Burden of Clinically Significant *Staphylococcus aureus* Infections and Predictors for Hospitalization for Skin and Soft Tissue Infections, Fulton County, Georgia, 2017

**DOI:** 10.1093/ofid/ofad601

**Published:** 2023-12-07

**Authors:** Katherine I Phillip, Andrew S Webster, Susan M Ray, Amber Britton, David Swerdlow, Scott K Fridkin

**Affiliations:** Department of Epidemiology, Rollins School of Public Health, Emory University, Atlanta, Georgia, USA; Division of Infectious Diseases, Department of Medicine, Emory School of Medicine, Atlanta, Georgia, USA; Department of Research, Atlanta Veterans Affairs Medical Center, Decatur, Georgia, USA; Georgia Emerging Infections Program, Decatur, Georgia, USA; Division of Infectious Diseases, Department of Medicine, Emory School of Medicine, Atlanta, Georgia, USA; Georgia Emerging Infections Program, Decatur, Georgia, USA; Division of Infectious Diseases, Department of Medicine, Emory School of Medicine, Atlanta, Georgia, USA; Department of Research, Atlanta Veterans Affairs Medical Center, Decatur, Georgia, USA; Georgia Emerging Infections Program, Decatur, Georgia, USA; Medical Development and Scientific/Clinical Affairs, Pfizer Vaccines, Collegeville, Pennsylvania, USA; Medical Affairs, HilleVax Inc, Boston, Massachusetts, USA; Division of Infectious Diseases, Department of Medicine, Emory School of Medicine, Atlanta, Georgia, USA; Georgia Emerging Infections Program, Decatur, Georgia, USA

**Keywords:** antibiotic resistance, MRSA, MSSA, *Staphylococcus aureus*

## Abstract

**Background:**

Incidence estimates of *Staphylococcus aureus* infections rarely include the full spectrum of clinically relevant disease from both community and healthcare settings.

**Methods:**

We conducted a prospective study capturing all *S aureus* infections in Fulton County, Georgia, during 2017. Medical records of patients with any incident infection (clinical cultures growing *S aureus* from any site, without prior positive culture in previous 14 days) were reviewed. Estimates of disease incidence were calculated using age-, race-, and sex-specific population denominators accounting for weighted sampling methods. Multivariable logistic regression models were used to identify risk factors for hospitalization among patients with skin and soft tissue infections (SSTIs).

**Results:**

The overall incidence of clinically relevant *S aureus* infection was 405.7 cases per 100 000 people (standard error [SE], 5.62 [range, 400.1–411.3]). Overall incidence for those of Black race was 500.84 cases per 100 000 people (SE, 14.55), whereas White patients had overall incidence of 363.67 cases per 100 000 people (SE, 13.8). SSTIs were the most common infection (2351; 225.8 cases per 100 000 people [SE, 7.1]), and 30% required hospitalization. Among SSTIs, after adjusting for invasive disease, cellulitis, diabetes, and demographics, independent predictors of hospitalization included methicillin-resistant *S aureus* (adjusted odds ratio [aOR], 1.6 [95% confidence interval {CI}, 1.0–2.7]) and homelessness (aOR, 4.9 [95% CI, 1.1–22]).

**Conclusions:**

The burden of clinically relevant *S aureus* infections is high, particularly among the Black population, and risks for hospitalization among SSTIs include isolate factors and factors related to patients’ vulnerability.

## Teaser

Burden estimates of *Staphylococcus aureus* infections frequently fail to cover the spectrum of disease. Skin/soft tissue infections are the most common *S aureus* infection and particularly affect the Black population. Risks for hospitalization include diabetes, homelessness, and methicillin-resistant *S aureus*.

Methicillin-resistant *Staphylococcus aureus* (MRSA) and methicillin-susceptible *S aureus* (MSSA) are common causes of infection both in community and hospital settings. Estimates of the burden of invasive MRSA infections in public health practice are performed annually by the Centers for Disease Control and Prevention (CDC) Emerging Infections Program (EIP); recent published national estimates include roughly 119 000 MRSA bloodstream infections (BSIs) in 2017 and 80 500 invasive MRSA infections in 2011 [1, 2]. Institution- or healthcare system–based studies have characterized risk factors, trends, and clinical presentations of specific syndromes of *S aureus* infection as well [1–5]. However, skin and soft tissue infections (SSTIs) are challenging to include in routine surveillance and robust estimates of disease are lacking because SSTI are typically noninvasive, widely prevalent, and often seen and treated in ambulatory care settings. Burden estimates of the spectrum of *S aureus* infections often rely on use of administrative data, which can result in biased estimates due to inaccuracies in the coding process designed to maximize revenue for hospitalizations rather than accuracy in case detection [6–9].

This study is a novel approach to estimating the burden and clinical characteristics of all clinically significant *S aureus* infections in a single county, as well as assessing predictors for hospitalization due to *S aureus* among clinically relevant SSTIs. Rather than relying on administrative data, we apply sampling and weighting to extrapolate data from medical record reviews and estimate exposures and clinical factors without assessment of each individual case, while still providing robust estimates of incidence and frequency.

## METHODS

### Study Design and Population

This a time-limited expansion to the existing Georgia EIP healthcare-associated infection laboratory-based surveillance for *S aureus* (funded by the CDC). EIP surveillance methods for population-based *S aureus* surveillance are described in detail elsewhere [10]. The expansion included a modification to case ascertainment, which normally allows for medical record review for only specimens sourced from normally sterile sites, to include any specimen source. The study population includes 1 041 432 people residing in Fulton County, Georgia in 2017; testing results from all 19 acute care hospitals, 1 pediatric hospital, and 2 referral laboratories that serve this catchment area were reviewed.

### Definitions

A case was defined as a resident of Fulton County, Georgia, from whom a specimen obtained for clinical (diagnostic) purposes grew *S aureus*. Residents included those receiving care in a hospital, residing in long-term care facilities, institutionalized, experiencing homelessness, or residing in private residences. Cases were categorized as MRSA or MSSA based on susceptibility test results from local clinical laboratories. Cases were classified as invasive *S aureus* if the positive culture was from a normally sterile site, such as the blood, cerebrospinal fluid, pleural fluid, pericardial fluid, peritoneal fluid, bone, joint or synovial fluid, and internal body sites (liver, heart, brain, kidneys, spleen, pancreas, ovary, lymph node, or vitreous fluid). If any positive culture was collected from a normally sterile site, the case was considered invasive regardless of other positive cultures from nonsterile sites in the same patient. Noninvasive cases were defined as cases with all positive cultures arising from a nonsterile site. Any case receiving treatment for the *S aureus* identified by culture was considered clinically significant.

Cases were further categorized based on timing of culture: hospital-onset (HO) if the patient had a positive culture collected >3 days after admission; or hospital-associated, community-onset (HACO) if the patient had prior healthcare exposures within the previous 12 months but not HO [10]. The remaining cases were community-associated, meaning the specimens were obtained as an outpatient or within the first 3 days of hospitalization, without any healthcare exposure in the previous 12 months.

### Sampling

All invasive cases received a full medical record review (not sampled). Noninvasive cases were randomly sampled (1:4) and received a full medical record review. Additional information was recorded based on invasive status, infection type, and outcome. Specimens obtained solely for the purpose of surveillance or testing of carriage were considered “surveillance testing” and were excluded prior to sampling. The total number of observations sampled was 1646 cases, comprised of 549 invasive and 1097 noninvasive cases. Notably, 15 noninvasive cases sampled for medical record review were determined to more appropriately classified as invasive cases, resulting in 60 additional (after applying weights) invasive cases for analysis (n = 619; Table 1).

**Table 1.** Incidence and Case Count by Demographic Category for Overall Infections and Skin and Soft Tissue Infections

Characteristic	Overall Infection (n = 4225)				SSTI (n = 2351)			
	No. (%)	SE	Incidence	SE	No. (%)	SE	Incidence	SE
Total	4225 (100)	55.043	405.69	5.29	2351 (100)	73.503	225.75	7.06
Sex								
Male	2237 (53)	68.031	443.49	13.49	1297 (55)	62.626	257.13	12.42
Female	1988 (47)	67.284	370.20	12.53	1054 (45)	57.998	196.27	10.80
Race								
White	1733 (47)	65.765	363.67	13.80	876 (37)	54.017	183.83	11.34
Black	2327 (55)	67.583	500.84	14.55	1374 (58)	63.73	295.73	13.72
Other	166 (3.9)	24.808	165.54	24.74	101 (4.3)	19.856	100.72	19.80
Age								
<18	616 (15)	45.916	265.27	19.77	507 (22)	42.934	218.33	18.49
18–39	1059 (25)	56.933	296.02	15.91	673 (29)	48.403	188.12	13.53
40–65	1606 (38)	62.941	481.94	18.89	826 (35)	52.634	247.87	15.79
≥65	944 (22)	50.877	798.50	43.04	345 (15)	34.813	291.83	29.45
Status								
Invasive	609 (14)	24.375	58.48	2.34	87 (3.7)	11.634	8.35	1.12
MRSA	1315 (31)	58.234	279.43	6.73	770 (33)	45.43	73.94	4.36

Errors are specific to each value and are based on standard sampling error. Incidence is per 100 000 people.

Abbreviations: MRSA, methicillin-resistant *Staphylococcus aureus*; SE, standard error; SSTI, skin and soft tissue infection.

### Clinical Data Abstraction

Demographics, underlying illness, treatments, and outcomes were abstracted using a standardized form by trained surveillance staff. Clinician-derived diagnoses were determined by mapping clinical documentation from treatment notes to predetermined clinical conditions associated with *S aureus* infection. Cases could have >1 clinical diagnosis. Only cases considered clinically significant (ie, received treatments such as antibiotics or drainage) were used in analysis, resulting in an unweighted sample size of 1453 patients.

### Missing Data

Race was abstracted from medical records, which relies on institution-specific practices at patient registration and was not standardized. Missing race data were imputed using a frequency matching technique to assign race to observations in proportion to the observed racial distribution of each stratum under consideration. Missing race observations (18% [301 observations]) were not random; 85% were from the 2 referral laboratories, which process mostly skin swabs. These observations were split into strata based on age, sex, and race; they were then randomly populated with Black, White, or other race values to match the frequency distribution of race in the corresponding strata among cases with known race. The process was repeated for missing ethnicity observations (20% [337 observations]) using frequency distribution of ethnicity by each age and sex strata in Fulton County [11]. This process assumes that the racial distribution of cases with unknown race is similar to the racial distribution of the cases with known race.

### Statistical Analyses

All analyses were performed on clinically significant cases exclusively. Survey analysis, including accounting for weights, generating values with error, and regression analysis were completed using the R package “survey” [12]. This package accounts for survey weights specified in the dataset, which were based on invasive versus noninvasive status. Invasive status infections were counted as-is with a weight of 1, while noninvasive infections were given a weight of 4 to account for the 1:4 sampling method. Incidence was calculated by key demographic and by infection type using case count point estimates and associated error generated by “survey” and respective 2017 US census denominators for Fulton County [11].

Descriptive epidemiologic analysis focused on the subset of SSTIs. To minimize information bias, cases with multiple clinician-derived diagnoses including SSTI and surgical site infection (SSI) (eg, overlap between SSTI and SSI) were omitted due to the inability to distinguish if the SSI was clinically the same as the SSTI or if they were separate infections.

Logistic regression was used to identify significant predictors of hospitalization among community-onset SSTI. Because most observations with missing race data were from SSTI observations, the SSTI cohort was limited to 1848 SSTIs that had no missing race data, and those that were not HO-SSTI. Models were built starting with factors that were statistically significant in univariate analysis (not shown) or deemed important for analysis and built forward to include factors of interest. Factors that were not statistically significant in the previous models were not carried on to further models. HO cases were omitted from regression analyses since all HO cases are hospitalized by definition. Statistical significance was defined as *P* ≤ .05.

Other analyses, data cleaning, and data visualization were completed using R statistical software (version 4.2.2) and other base R packages including “dplyr” and “tidyverse.” This research has been approved by the institutional review boards at the Atlanta Veterans Affairs Medical Center (data collection) and Emory University (data analysis).

## RESULTS

### Estimates of Incidence

After weights were applied, there were 4225 (standard error [SE], 55.04) cases of clinically relevant *S aureus* in Fulton County in 2017, comprised of 609 invasive and 3616 noninvasive cases (SE, 24.38 and 73.951, respectively). Of the 4225 cases, 31% (1315) were MRSA. Invasive MRSA included 243 cases (SE, 15.83), 40% of all invasive cases.

**Figure 1.**
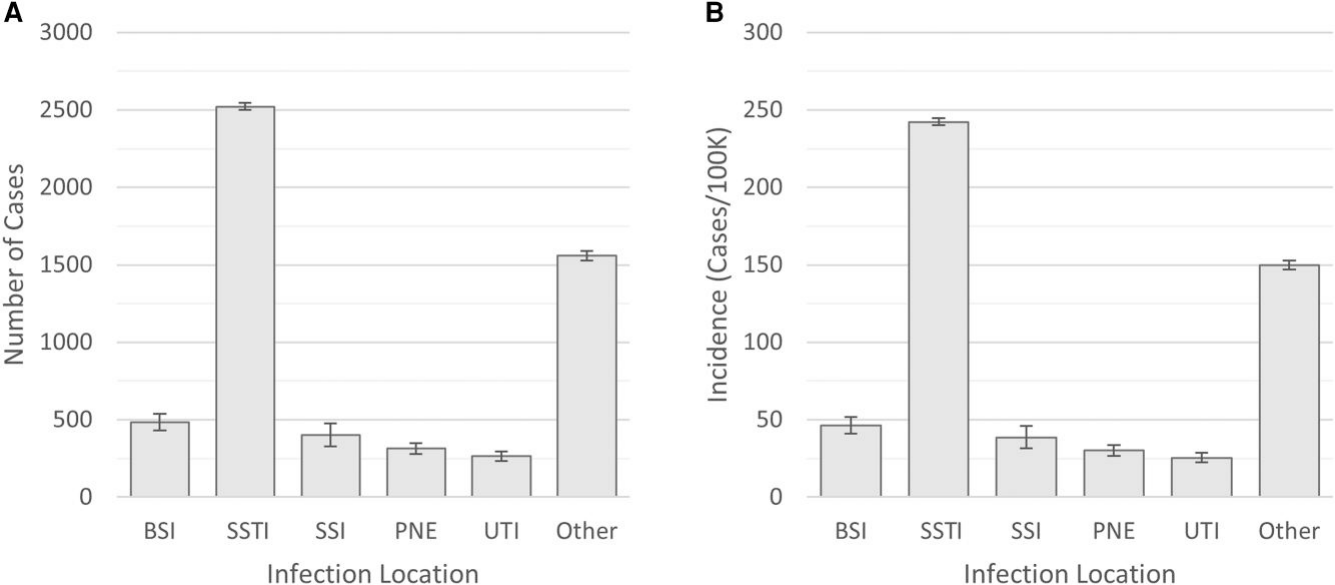
Count and incidence of clinically relevant *Staphylococcus aureus* cases by infection location in Fulton County, Georgia, 2017. This figure shows counts (*A*) and incidence (*B*) by infection location after survey weighting has been taken into account. Overall count of cases was 4225 (standard error [SE], 55). Overall incidence was 405.7 (SE, 5.3). Error bars are specific to each infection location count and incidence and error values are based on standard sampling error. Abbreviations: BSI, bloodstream infection; PNE, *Staphylococcus aureus* pneumonia; SSI, surgical site infection; SSTI, skin and soft tissue infection; UTI, urinary tract infection.

Incidence of clinically relevant *S aureus* infection was 405.7 cases per 100 000 people (SE, 5.62 [range, 400.1–411.3]) (Table 1), and invasive infections of all types had an incidence of 58.5 cases per 100 000 people (SE, 2.34). Overall MRSA and MSSA infections had respective incidence of 122.27 (SE, 5.59) and 270.43 (SE, 6.73) per 100 000 people. Most common sites included BSI, SSTI, SSI, *S aureus* pneumonia, and urinary tract infection (UTI); rates are illustrated in Figure 1. SSTIs were the most common type of infection (Table 1), and 33% of SSTIs were MRSA. Invasive SSTI was rare, roughly 3% of all SSTIs, with a rate of 8.35 per 100 000 people (SE, 1.12).

Incidence rates of clinically relevant *S aureus* infections were highest among the Black population, with 500.84 cases per 100 000 people (SE, 14.55) for overall infections and 295.73 SSTI cases per 100 000 people (SE, 13.72) (Table 1). White patients had overall infection incidence of 363.67 per 100 000 people (SE, 13.8) and 183.83 SSTI cases per 100 000 people (SE, 11.34). Other races had 165.54 overall infections per 100 000 people (SE, 24.74) and 100.72 SSTI cases per 100 000 people (SE, 19.8). Males had higher incidence compared with females for both infection categories. In overall infections, incidence increases as age increases. However, for SSTI, the lowest incidence is found in those aged 18–39 years, followed by those aged <18 years. The proportion of cases caused by MRSA was consistent between overall infection (31% of cases) and SSTI (33% of cases).

### Clinical Characteristics of SSTI Cases

Assessment of clinical presentation of SSTIs show that 3.7% had an invasive component, and 48% of presentations included abscess and 30% of presentations included cellulitis (Table 2). Uncommon infection types included mastitis and myositis. Community-associated infections comprised the majority of all infection types, excluding chronic wounds/decubitus ulcers and necrotizing fasciitis; for both of these syndromes, 50% of cases were HACO infections. HO infections are highest in burn-related infections, chronic wound/decubitus ulcers, and cellulitis, making up 33%, 10%, and 4.6% of respective infections (Table 2). Among all SSTIs, 702 (30%) were hospitalized and 49% received drainage as part of their treatment.

**Table 2.** Clinical Presentation of Skin and Soft Tissue Infections (n = 2347)

Infection Type	No. (%)	SE	HO Infection, No.	HACO Infection, No.	CA Infection, No.
Abscess	1132 (48)	60.4	16	172	944
Acute infected wound	380 (16)	37.7	12	116	252
Pustule or minor skin infection	404 (17)	38.8	0	68	336
Cellulitis	696 (30)	49.5	32	156	508
Chronic wound or decubitus ulcer infected	324 (14)	34.9	32	164	128
Necrotizing fasciitis	32 (1.4)	11.3	4	16	12
Burn related	12 (0.5)	6.9	4	4	4
Herpes zoster related	8 (0.3)	5.7	0	0	8
Mastitis	4 (0.2)	4	0	0	4

Cases can have >1 presentation form. Percentage of counts were taken from total number of cases, not number of presentations. There were 2996 clinical presentations recorded.

Abbreviations: CA, community-associated; HACO, hospital-associated, community-onset; HO, hospital-onset; SE, standard error.

### Predictors of Hospitalization Among SSTIs

Initial evaluation for predictors of hospitalization focused on isolate, patient, and infection characteristics. As expected, diabetes, recent healthcare exposure (likely a proxy for underlying illness), and severity of infection (ie, invasive nature, component of cellulitis, or necrotizing fasciitis) are associated with hospitalization (Table 3, model 1). Persons with infections from MRSA were more likely to be hospitalized (adjusted odds ratio [aOR], 1.8 [95% confidence interval {CI}, 1.2–2.9]; *P* = .01) and those with Black race were borderline more likely to be hospitalized (aOR, 1.49 [95% CI, .9–2.5]; *P* = .13) (Table 3).

**Table 3.** Logistic Regression for Hospitalization Among 1848 *Staphylococcus aureus* Skin and Soft Tissue Infections (With Known Race Values)

Characteristic	Model 1			Model 2		
	OR	(95% CI)	*P* Value	OR	(95% CI)	*P* Value
MRSA	1.64	(1.01–2.65)	.046	1.57	(.95–2.59)	.077
Invasive	18.6	(4.72–73.5)	<.001	19.4	(4.80–78.1)	<.001
Cellulitis/necrotizing fasciitis	2.76	(1.66–4.60)	<.001	2.59	(1.55–4.33)	<.001
Healthcare exposure previous 12 mo	4.13	(2.35–7.26)	<.001	…	…	…
Black race	1.49	(.89–2.49)	.13	1.49	(.86–2.47)	.2
Female sex	0.72	(.45–1.16)	.2	0.76	(.47–1.23)	.3
Age, y						
0–17	…	…		…	…	…
18–39	2.58	(1.13–5.87)	.024	2.57	(1.13–5.88)	.025
40–64	2.08	(.89–4.91)	.093	1.97	(.83–4.67)	.12
≥65	1.88	(.66–5.35)	.2	1.67	(.56–5.00)	.4
Diabetes	3.53	(2.00–6.24)	<.001	3.65	(2.03–6.55)	<.001
Hospitalization in previous 12 mo	…	…		4.85	(2.60–9.07)	<.001
Homeless	…	…		4.86	(1.10–21.5)	.037
LTC stay in previous 12 mo	…	…		1.49	(.32–6.87)	.6

Hospital-onset cases are excluded from this analysis. Model 1 focused on patient demographic, pathogen, and clinical presentation; model 2 added alternative healthcare or other high-risk exposures rather than summary healthcare exposure variable.

Abbreviations: CI, confidence interval; LTC, long-term care; MRSA, methicillin-resistant *Staphylococcus aureus*; OR, odds ratio.

To identify if more specific exposures would offset the association observed with MRSA, we explored model 2 to evaluate hospitalization in the last 12 months and homelessness as specific exposures in place of “any” healthcare exposure. Both these new exposure variables were significantly associated with hospitalization and did not reduce the impact of MRSA status on the likelihood of hospitalization appreciably (Table 3). Model 2 also evaluated long-term care residence in any of the previous 12 months, which was not predictive of hospitalization.

## DISCUSSION

Incidence of clinically relevant *S aureus* infection is high in Fulton County, Georgia, at approximately 400 infections per 100 000 persons; furthermore, incidence was roughly 40% higher among Black residents than among White residents. Utilizing a sampling methodology to allow critical review of medical records, we estimated syndrome-specific rates of infection for the population as well, which can be used for future modeling exercises to ascertain potential impact of infection prevention efforts on disease-specific incidence from a population perspective, and not per hospital discharge. Use of *International Classification of Diseases*, *Tenth Revision* or *Ninth Revision*–coded data for *S aureus* is limited to estimates of sepsis or pneumonia for syndrome-specific disease; our estimates are precise and cover more types of *S aureus* infections considered clinically relevant. Also, our disease-specific incidence estimates were not dependent on patients being hospitalized, which is a limitation of most recent published disease burden estimates focused on sepsis, pneumonia, or any *S aureus* “not otherwise specified” [8].

From this population perspective, incidence of *S aureus* pneumonia and UTI is not substantially less than the better studied SSI or BSI and should not be ignored when considering disease burden; however, clinically speaking, it is difficult to distinguish between contamination, colonization, or infection or seeding from another site in pneumonia or UTI. Because SSTI is the most common presentation of clinically relevant *S aureus* infection and difficult to study in burden estimates, we describe the spectrum of presentations in this population. Standing out was the observation that 30% of SSTIs presenting with some component of cellulitis, although our estimate is limited by the unreliable and subjective documentation of skin appearance in medical records. The fact that a swab was submitted for culture suggests pus was present to culture, which is a hallmark of *S aureus* infection. About 30% of patients with SSTI were hospitalized, and predictors of hospitalization included MRSA infection after adjustment of type of infection, underlying illness, and healthcare exposures. We also identified that persons who are unhoused are at extreme risk for hospitalization, perhaps due to poor access to reliable care, influencing providers’ decisions to hospitalize the patient. However, the observation that Black residents may have a higher risk for hospitalization as well as higher risk for overall disease incidence reinforces the presence of racial disparities in *S aureus* infections [13–15].

There are several limitations of this study. All values presented in this study are based on estimates with error as this study utilized a sample of cases, and error is produced when analyzing and extrapolating the sample data. Although this method allows for precise estimation of incidence of common presentations of disease, rarer presentations may not be sampled. However, for large-scale, population-level incidence and diagnosis information, sampling cases provides a reliable method of collecting information without reliance on administrative data. Each case in this analysis could have multiple infection locations and clinical presentations with some inaccurate mapping of disease type to our clinician-derived diagnosis. We attempted to limit this with the SSTI analysis by removing potential overlap with SSI. Estimates of race-specific SST incidence are additionally limited as unknown race values were assigned based on distributions among known races and assumed infection types were distributed evenly between race categories, which may have contributed to a more conservative incidence estimate.

This study population was comprised of individuals residing in in Fulton County, Georgia, a large and diverse urban city area of Atlanta. The population studied is not representative of the state of Georgia or other areas in the United States, particularly in regard to race [16]. However, we provide race, sex, and age strata specific incidence as an effort to allow these values to be compared to other populations studied, and for use by modelers to have stratum-specific estimates of clinically relevant *S aureus* infection and SSTI specifically.

Our study demonstrated that the burden of all *S aureus* infections is higher than that of estimates based on only MRSA, invasive, or hospitalized patients. Our estimates are substantially lower (roughly 50%) than 2008 estimates derived from the Veterans Administration database in Baltimore, Maryland, perhaps reflecting an older population in that report and national trends at reductions in incidence of *S aureus* infections since then [2, 9]. Findings from our study are more updated and can help inform efforts to estimate potential reductions in disease burden for novel intervention strategies that target specific populations with potential vaccines or skin decontamination strategies. *Staphylococcus aureus* continues to affect broad populations with a wide variety of presentations, but disparities among racial groups and healthcare exposures deserve urgent attention.

## Notes

***Acknowledgments.*** We acknowledge the work of the Georgia Emerging Infections Program (EIP) and staff who collected and maintained the invasive *S aureus* data.

***Author contributions.*** K. I. P. and S. K. F. performed drafting of the manuscript and data analysis. S. K. F., A. B., and A. S. W. performed data acquisition, methodology/design, and manuscript revision. S. M. R., D. S., and S. K. F. performed concept, support, and supervision. K. I. P., A. S. W., S. M. R., A. B., D. S., and S. K. F. provided critical review and approval of the manuscript.

***Patient consent.*** This study does not include factors necessitating patient consent.

***Financial support.*** This work was supported by the Centers for Disease Control and Prevention EIP (grant number U50CK000485), which supported the Georgia EIP surveillance of invasive *S aureus*. Surveillance of noninvasive *S aureus* was funded through a research agreement between Pfizer, Inc and Emory University (agreement number CP154653).
